# Quantitative assessment of background parenchymal enhancement is associated with lifetime breast cancer risk in screening MRI

**DOI:** 10.1007/s00330-024-10758-9

**Published:** 2024-04-29

**Authors:** Ran Yan, Wakana Murakami, Shabnam Mortazavi, Tiffany Yu, Fang-I. Chu, Stephanie Lee-Felker, Kyunghyun Sung

**Affiliations:** 1 Department of Radiological Sciences, David Geffen School of Medicine, University of California, Los Angeles, CA USA; 2 Department of Bioengineering, Henry Samueli School of Engineering, University of California, Los Angeles, CA USA; 3 Department of Radiology, Showa University Graduate School of Medicine, Tokyo, Japan; 4 Department of Radiation Oncology, University of California, Los Angeles, CA USA

**Keywords:** Background parenchymal enhancement, Breast cancer, Quantitative background parenchymal enhancement, Lifetime risk, *BRCA* germline mutation

## Abstract

**Objectives:**

To compare the quantitative background parenchymal enhancement (BPE) in women with different lifetime risks and *BRCA* mutation status of breast cancer using screening MRI.

**Materials and methods:**

This study included screening MRI of 535 women divided into three groups based on lifetime risk: nonhigh-risk women, high-risk women without *BRCA* mutation, and *BRCA1/2* mutation carriers. Six quantitative BPE measurements, including percent enhancement (PE) and signal enhancement ratio (SER), were calculated on DCE-MRI after segmentation of the whole breast and fibroglandular tissue (FGT). The associations between lifetime risk factors and BPE were analyzed via linear regression analysis. We adjusted for risk factors influencing BPE using propensity score matching (PSM) and compared the BPE between different groups. A two-sided Mann–Whitney U-test was used to compare the BPE with a threshold of 0.1 for multiple testing issue-adjusted *p* values.

**Results:**

Age, BMI, menopausal status, and FGT level were significantly correlated with quantitative BPE based on the univariate and multivariable linear regression analyses. After adjusting for age, BMI, menopausal status, hormonal treatment history, and FGT level using PSM, significant differences were observed between high-risk non-*BRCA* and *BRCA* groups in PE_FGT_ (11.5 vs. 8.0%, adjusted *p* = 0.018) and SER_FGT_ (7.2 vs. 9.3%, adjusted *p* = 0.066).

**Conclusion:**

Quantitative BPE varies in women with different lifetime breast cancer risks and *BRCA* mutation status. These differences may be due to the influence of multiple lifetime risk factors. Quantitative BPE differences remained between groups with and without *BRCA* mutations after adjusting for known risk factors associated with BPE.

**Clinical relevance statement:**

*BRCA* germline mutations may be associated with quantitative background parenchymal enhancement, excluding the effects of known confounding factors. This finding can provide potential insights into the cancer pathophysiological mechanisms behind lifetime risk models.

**Key Points:**

- *Expanding understanding of breast cancer pathophysiology allows for improved risk stratification and optimized screening protocols.*
- *Quantitative BPE is significantly associated with lifetime risk factors and differs between BRCA mutation carriers and noncarriers.*
- *This research offers a possible understanding of the physiological mechanisms underlying quantitative BPE and BRCA germline mutations.*

## Introduction

Breast cancer is the most prevalent noncutaneous malignancy among women and is ranked as the second leading cause of cancer-related deaths [1]. The standard imaging tool for breast cancer screening is digital mammography. However, mammography sensitivity may be compromised in women with dense-breast tissue, as the masking effect of such tissue can obscure potential malignancies [2]. As a result, the American Cancer Society recommends that women with a lifetime risk exceeding 20% of developing breast cancer undergo breast magnetic resonance imaging (MRI) screening in addition to mammography [3]. MRI and mammography combined screening improves breast cancer survival in these individuals [4].

Predicting breast cancer risk would enhance patient stratification and lead to tailored screening tactics. Established breast cancer risk assessment models such as the Gail, Claus, and Tyrer-Cuzick models are widely utilized for stratifying patients into different risk groups but offer moderate predictive accuracy at the individual level [5–10]. There is an ongoing interest in the physiological mechanisms underlying these risk models. One potential factor is fibroglandular tissue (FGT) enhancement after contrast injection, i.e., background parenchymal enhancement (BPE). Previous studies have revealed a possible correlation between BPE and breast cancer risk [11–15], although this relationship remains controversial [16, 17]. Potential variations in BPE’s vascular and molecular characteristics may account for differences in breast cancer risk [18]. It also remains uncertain whether quantitative BPE characteristics significantly differ between women with and without a high lifetime risk. Such studies may uncover the role of BPE as an underlying physiological factor behind the classical risk models.

The *BRCA1* and *BRCA2* genes, known as tumor suppressor genes, encode proteins essential for DNA repair. Germline mutations in the *BRCA1/2* increase the risk of several cancers, most notably breast and ovarian cancers [19]. Comparison of BPE kinetic properties between *BRCA* mutation carriers and noncarriers could help explain the impact of *BRCA* on breast physiology, thereby further improving the diagnostics accuracy of MRI in populations at high risk of breast cancer. While previous studies have reported a lower BPE level in *BRCA1/2* mutation carriers in high-risk women using qualitative and quantitative methods [18, 20, 21], Goodburn et al. [22] has presented contrasting findings by showing no differences in BPE between carriers and noncarriers. BPE level is known to be hormone-sensitive and is associated with menstrual cycle, age, menopausal status, corresponding to hormonal changes, and breast density [23, 24], which may contribute to the conflicting results.

The variability in radiologist-assigned BPE categories within and between readers highlights the necessity for the quantitative study of BPE. Therefore, our study aimed to compare quantitative BPE measurements based on screening MRI between radiologically normal women with and without high lifetime risk and between *BRCA* mutation carriers and noncarriers. We determined which clinical factors in classical risk models are associated with quantitative BPE measurements. In addition, we investigated whether differences in BPE remained after adjusting for clinical factors that might influence BPE.

## Materials and methods

### Patient population

**Fig. 1:**
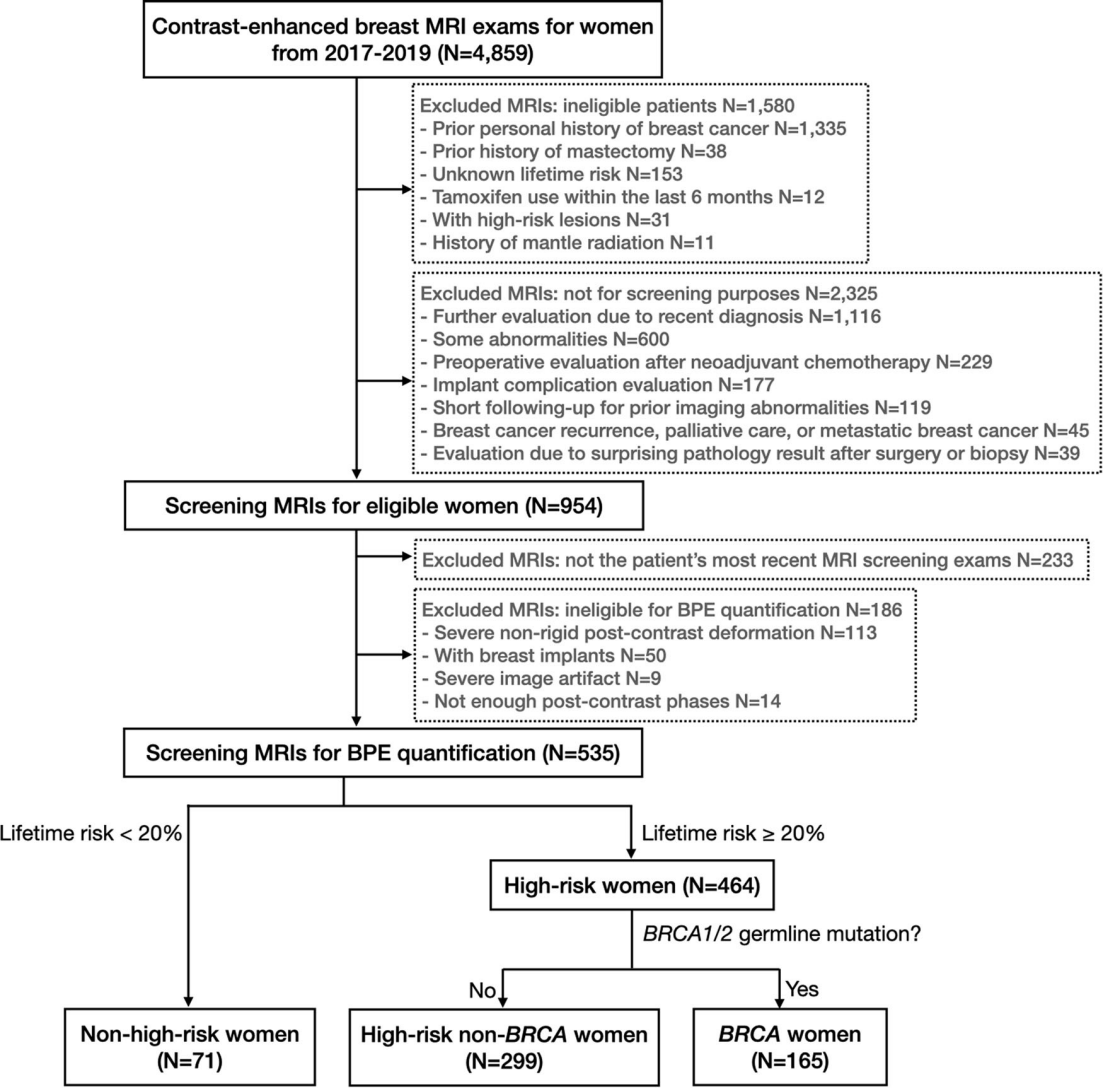
Flowchart of study sample selection

Our retrospective study was approved by the Institutional Review Board (IRB) and was conducted in compliance with the Health Insurance Portability and Accountability Act (HIPAA). IRB waived the requirement to obtain informed consent. We reviewed 4859 contrast-enhanced bilateral breast MRI exams for women between January 2017 and December 2019. Exclusions were made for patients with prior breast cancer, mastectomy history, unknown lifetime risk scores, and tamoxifen use within the last 6 months. The MRI exams that were not for screening purposes or not eligible for BPE quantification were further excluded. Further exclusion details are in Fig. 1. Premenopausal women underwent MRI screenings during the second week of their menstrual cycle to minimize the amount of estrogen-induced BPE [25].

The following characteristics were collected for eligible women based on the records at the MRI exam: age, body mass index (BMI), menopausal status, personal history of hormonal therapy within six months before MRI, and genetic test results. Breast density was derived from the MRI report as four FGT levels (almost entirely fat, scattered FGT, heterogeneous FGT, and extreme FGT). Menopausal status was determined based on patient-submitted questionnaires. Two breast fellowship-trained radiologists evaluated BPE levels (W.M. and S.M.). The Tyrer-Cuzick model [7] was used to calculate the lifetime risk score. Based on these scores and *BRCA1/2* mutation status, the eligible women were stratified into three groups: (1) nonhigh-risk group including women with lifetime risk < 20%; (2) high-risk non-*BRCA* group including women with 20% or higher lifetime risk without *BRCA1/2* mutation; (3) *BRCA* group including women with *BRCA1/2* mutation. Including nonhigh-risk women who underwent MRI scans could be attributed to the primary care providers’ limited familiarity with the latest screening guidelines or the patient’s personal choice for more comprehensive testing. Women with high-risk lesions and those with mantle field irradiation due to Hodgkin’s lymphoma were later eliminated from the nonhigh-risk group. The complete flowchart of the study is presented in Fig. 1.

### MRI protocols

MRI scans were performed in the prone position in the axial plane on a 3-T scanner (Siemens Verio, Erlangen, Germany). Image sequences included a T1-weighted non-fat-suppressed (T1-NFS) image and a T1-weighted fat-suppressed dynamic contrast-enhanced (DCE) MRI series with one precontrast and four postcontrast images. Gadolinium-based contrast (Magnevist, Bayer, Leverkusen, Germany) was administered at 0.1 mmol/kg, 2 mL/s, followed by a 20 mL saline flush. The first postcontrast sequence was acquired 120 s after the precontrast sequence, with other postcontrast sequences acquired every 90 s. The breast MRI protocol details are in Supplementary Material S1.

### FGT and BPE quantification

**Fig. 2:**
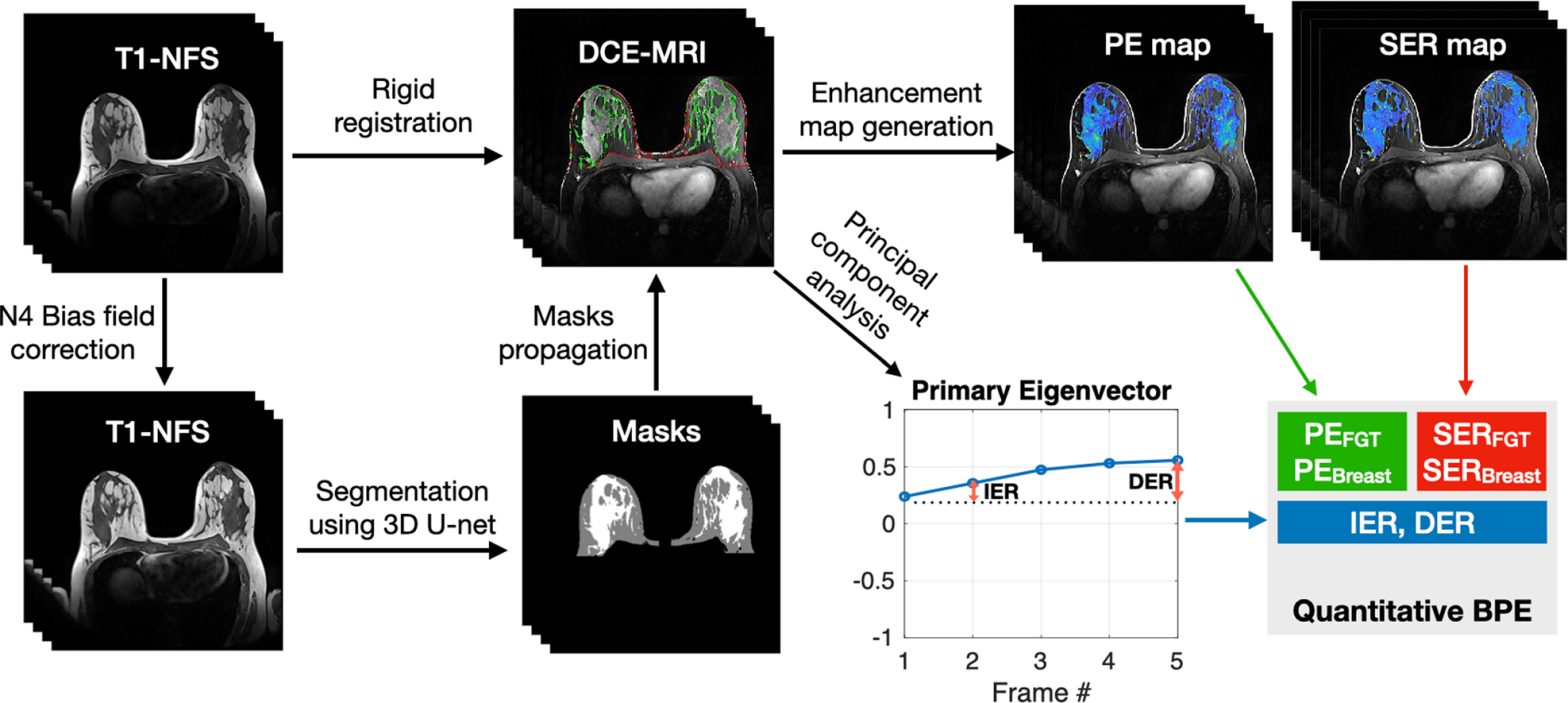
Overview of BPE quantification process. The three-dimensional volumes of breast and FGT were segmented on T1-NFS images. The segmented masks of the breast (*red outlines*) and FGT (*green*) were then transferred to the DCE-MRI. Enhancement maps, including the PE map and SER map, were generated based on the masks from which PE_FGT_, PE_Breast_, SER_FGT_, and SER_Breast_ were derived. The primary eigenvector of DCE-MRI was used to calculate another two BPE measurements, i.e., IER and DER. DCE dynamic contrast-enhanced, PE percent enhancement, SER signal enhancement ratio, IER initial enhancement ratio, DER delayed enhancement ratio

We used a fully automated method modified from a previous publication [26], to segment the whole breast and FGT. As shown in Fig. 2, after the N4 bias field correction [27], the entire breast and FGT three-dimensional volumes were segmented using a 3D U-net deep learning model on T1-NFS images. After applying image rigid registration, the segmented masks were transferred to the DCE-MRI series. The anterior border of the pectoralis muscle was defined as the edge between the breast and chest. The nipple and skin were excluded from the whole breast segmented masks. Vessels were excluded from the FGT segmentation masks if they were visible.

We measured PE, representing wash-in enhancement characteristics, and SER, representing delayed enhancement characteristics, over the FGT volume (FGT-wise BPE) and the whole breast volume (breast-wise BPE). The four quantitative BPE measurements were denoted as PE_FGT_, PE_Breast_, SER_FGT_, and SER_Breast_. The enhancement ratio threshold for each voxel in the PE map and SER map were set as 30% and 90%, respectively, referring to previous studies [28, 29]. The details of these four quantitative BPE calculations are explained in Supplementary Material S2. We also measured the IER and DER of the BPE based on the three-dimensional volume of the FGT. We applied the principal component analysis (PCA) method [18, 25] to the DCE-MRI image series. The principal eigenvector with the highest eigenvalue captures the maximum signal fluctuation from enhancing tissue in the FGT volume, which is supposed to reflect BPE kinetics. Therefore, based on the principal eigenvector, IER was defined as the percent increase of postcontrast 120-s early phase compared to precontrast, and DER was the percent increase of postcontrast 390-s delayed-phase compared to precontrast, as shown in Fig. 2. BPE quantification was performed using MATLAB (MathWorks, Natick, MA).

### Statistical analyses

We reported descriptive statistics for study group characteristics. We conducted two comparisons: high-risk non-*BRCA* vs. nonhigh-risk (among women without *BRCA1/2* mutations) and high-risk non-*BRCA* vs. *BRCA* (among high-risk women). Ages and BMI were compared using the Mann–Whitney U-test, while menopausal status, hormonal treatment history, FGT level, and BPE level were compared using the chi-squared test with a significance level of 0.05.

Furthermore, corresponding to the data distribution, two-sided Mann–Whitney U-tests were used to compare the BPE measurements. To account for multiple testing issues, Bonferroni procedure was applied to correct the significance level for the number of hypothesis testing, m, with the family-wise error rate (FWER) at threshold of 0.1. The patients were further divided into subcohorts based on breast density and menopausal status. “Dense-breast women” includes women with heterogeneous and extreme FGT. Quantitative BPE was compared within the subcohorts, and adjusted *p* values, as unadjusted *p* value/m, were reported.

We used univariate and multivariable linear regression analysis to evaluate the association between BPE measurements and clinical factors, including age, BMI, menopausal status, hormonal treatment, FGT level, and *BRCA* gene mutation status. We reported correlation coefficients with 95% confidence intervals and corresponding *p* values with a significance level of 0.05.

Since BPE is sensitive to endogenous hormonal changes and other factors [23, 24], we used propensity score matching (PSM) to control for confounders [30], including age, BMI, menopausal status, hormonal treatment history, and FGT level. PSM was performed twice using nearest-neighbor matching at a 1:1 ratio, first matching nonhigh-risk (*N* = 71) to high-risk non-*BRCA* and then *BRCA* (*N* = 165) to high-risk non-*BRCA*. We reported patient characteristics after PSM and compared BPE measurements in matched groups.

We tested the reliability of our results by examining the effect of BPE thresholds and early phase selection on BPE differences. PE and SER were estimated using thresholds of 10–90% in 10% increments and different postcontrast phases as the early phase. We compared BPE between risk groups using the Mann–Whitney U-test for each threshold and phase. We reported unadjusted *p* values with a significance level of 0.05. Statistical analyses were performed using Python’s library SciPy (version 1.9.3) and R (version 4.1.2).

## Results

The final cohort includes 535 eligible patients (high-risk non-*BRCA*, 299 patients; nonhigh-risk, 71 patients; *BRCA*, 165 patients). The clinical and radiographic characteristics of the study cohort are summarized in Table 1. There were significant differences in age, menopause status, FGT level, and BPE level distribution in the pairwise comparison of the three groups. The BMI significantly differed between high-risk non-*BRCA* and *BRCA* groups (unadjusted *p* = 0.018).

**Table 1:** Characteristics of study subjects

	High-risk non-*BRCA*	Nonhigh-risk	Unadjusted *p*	High-risk non-*BRCA*	*BRCA*	Unadjusted *p*
No. of patients	299	71		299	165	
Age (median (range))	46 (23–76)	56 (30–86)	< 0.001*	46 (23–76)	40 (21–83)	0.005*
BMI (median (range))	24.1 (16.5–48.7)	24.1 (18.7–40.7)	0.70	24.1 (16.5–48.7)	25.1 (17.4–44.4)	0.018*
Menopausal status			< 0.001*			0.02*
Premenopausal	198 (66.2%)	19 (26.8%)		198 (66.2%)	91 (55.2%)	
Postmenopausal	101 (33.8%)	52 (73.2%)		101 (33.8%)	74 (44.8%)	
Hormone treatment			0.20			0.08
Yes	72 (24.1%)	23 (32.4%)		72 (24.1%)	53 (32.1%)	
No	227 (75.9%)	48 (67.6%)		227 (75.9%)	112 (67.9%)	
FGT			0.002*			0.03*
Almost entirely fat	7 (2.3%)	4 (5.6%)		7 (2.3%)	10 (6.1%)	
Scattered	80 (26.8%)	27 (38.0%)		80 (26.8%)	55 (33.3%)	
Heterogeneous	114 (38.1%)	32 (45.1%)		114 (38.1%)	61 (37.0%)	
Extreme	98 (32.8%)	8 (11.3%)		98 (32.8%)	39 (23.6%)	
BPE			< 0.001*			< 0.001*
Minimal	61 (20.4%)	34 (47.9%)		61 (20.4%)	72 (43.6%)	
Mild	96 (32.1%)	17 (23.9%)		96 (32.1%)	55 (33.3%)	
Moderate	96 (32.1%)	20 (28.2%)		96 (32.1%)	33 (20.0%)	
Marked	46 (15.4%)	0 (0.0%)		46 (15.4%)	5 (3.0%)	

The *p* values are from the Mann–Whitney U-test for age and BMI and chi-squared test for menopausal status, hormone treatment history, FGT, and BPE

*Significant difference with unadjusted *p* value < 0.05

### Comparisons of quantitative BPE for all women

**Fig. 3:**
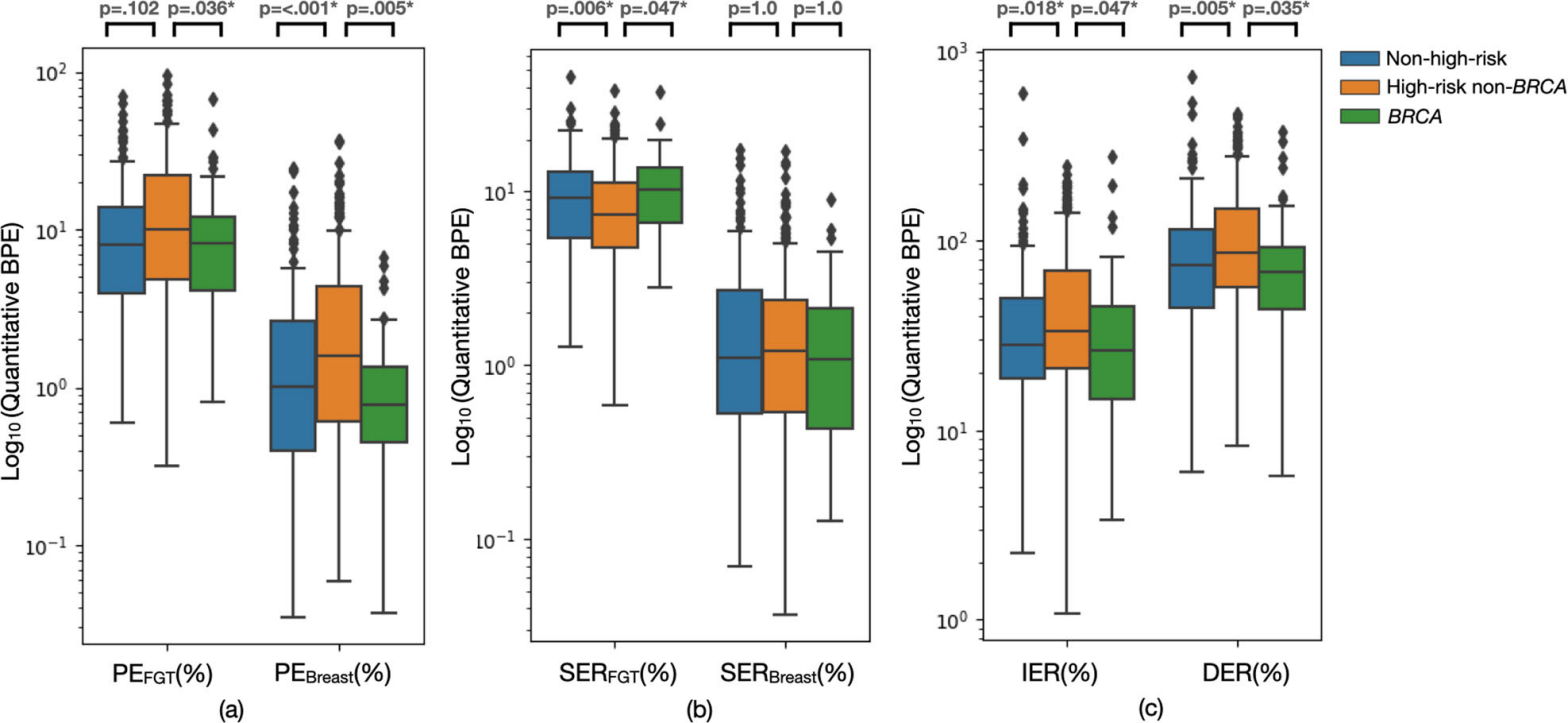
Boxplots of quantitative BPE comparison with adjusted *p* values using Mann–Whitney U-test. **a** Comparison of PE. **b** Comparison of SER. **c** Comparison of IER and DER. *Significant difference was identified as adjusted *p* < 0.10 (pre-specified threshold of FWER)

As shown in Table 2, after adjusting for multiple testing issues, for high-risk non-*BRCA* vs. nonhigh risk, we found significant differences in PE_breast_ (1.6 vs. 0.8%, adjusted *p* < 0.001), SER_FGT_ (7.4 vs. 10.2%, adjusted *p* = 0.006), IER (33.5 vs. 26.4%, adjusted *p* = 0.018), and DER (85.6 vs. 68.4%, adjusted *p* = 0.005). For high-risk non-*BRCA* vs. *BRCA*, we found significant differences in PE_FGT_ (10.1 vs. 8.0%, adjusted *p* = 0.036), PE_breast_ (1.6 vs. 1.0%, adjusted *p* = 0.005), SER_FGT_ (7.4 vs. 9.3%, adjusted *p* = 0.047), IER (33.5 vs. 28.2%, adjusted *p* = 0.047), and DER (85.6 vs. 73.9%, adjusted *p* = 0.035). Figure 3 provides a detailed illustration of BPE data distributions with adjusted *p* values.

**Table 2:** Quantitative BPE comparison in all women using Mann–Whitney U-test

	High-risk non-*BRCA*	Nonhigh-risk	Adjusted *p*	High-risk non-*BRCA*	*BRCA*	Adjusted *p*
PE_FGT_ (%) (Median [IQR])	10.1 (17.3)	8.2 (8.0)	0.102	10.1 (17.3)	8.0 (9.8)	0.036*
PE_Breast_ (%) (Median [IQR])	1.6 (3.7)	0.8 (0.9)	< 0.001*	1.6 (3.7)	1.0 (2.2)	0.005*
SER_FGT_ (%) (Median [IQR])	7.4 (6.5)	10.2 (7.2)	0.006*	7.4 (6.5)	9.3 (7.6)	0.047*
SER_Breast_ (%) (Median [IQR])	1.2 (1.8)	1.1 (1.7)	1.0	1.2 (1.8)	1.1 (2.2)	1.0
IER (%) (Median [IQR])	33.5 (48.0)	26.4 (30.2)	0.018*	33.5 (48.0)	28.2 (31.2)	0.047*
DER (%) (Median [IQR])	85.6 (91.2)	68.4 (48.2)	0.005*	85.6 (91.2)	73.9 (71.1)	0.035*

*IQR* interquartile range

*Significant difference was identified as adjusted *p* value < 0.10 (pre-specified threshold of FWER)

Note: number of hypothesis testing = 6, adjusted *p* value = unadjusted *p* value / 6

### Comparisons of quantitative BPE for subcohorts

**Fig. 4:**
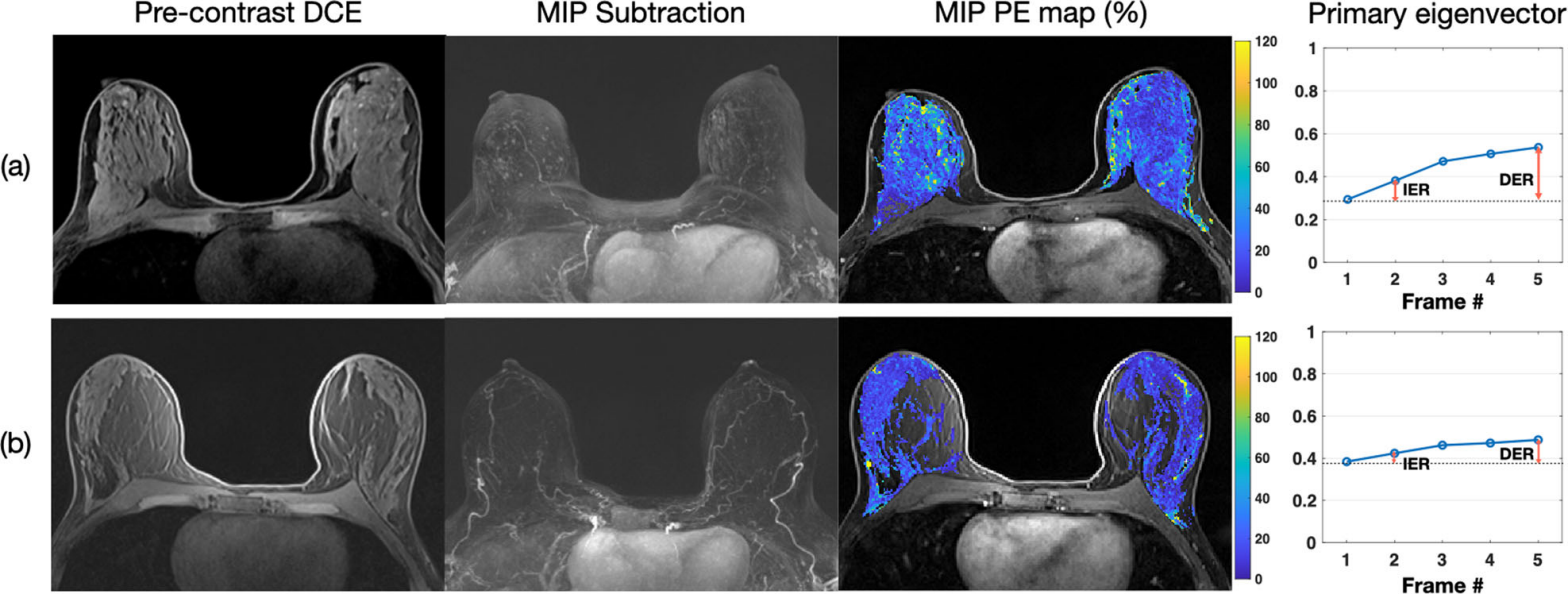
MRIs and BPE of two representative dense-breast normal-weight postmenopausal women with (**a**) high-risk non-*BRCA* mutation and (**b**) nonhigh-risk. MRI images include precontrast DCE-MRI, MIP of first postcontrast subtraction DCE-MRI, MIP of ten slices of PE map, and the primary eigenvector used to measure IER and DER. **a** A 49-year-old woman with a lifetime risk of 45.0% and a BMI of 22.45. FGT level is extreme fibroglandular tissue, and BPE level is marked. **b** A 53-year-old woman with a lifetime risk of 7.7% and a BMI of 20.08. FGT level is heterogeneously fibroglandular tissue, and BPE level is minimal

**Fig. 5:**
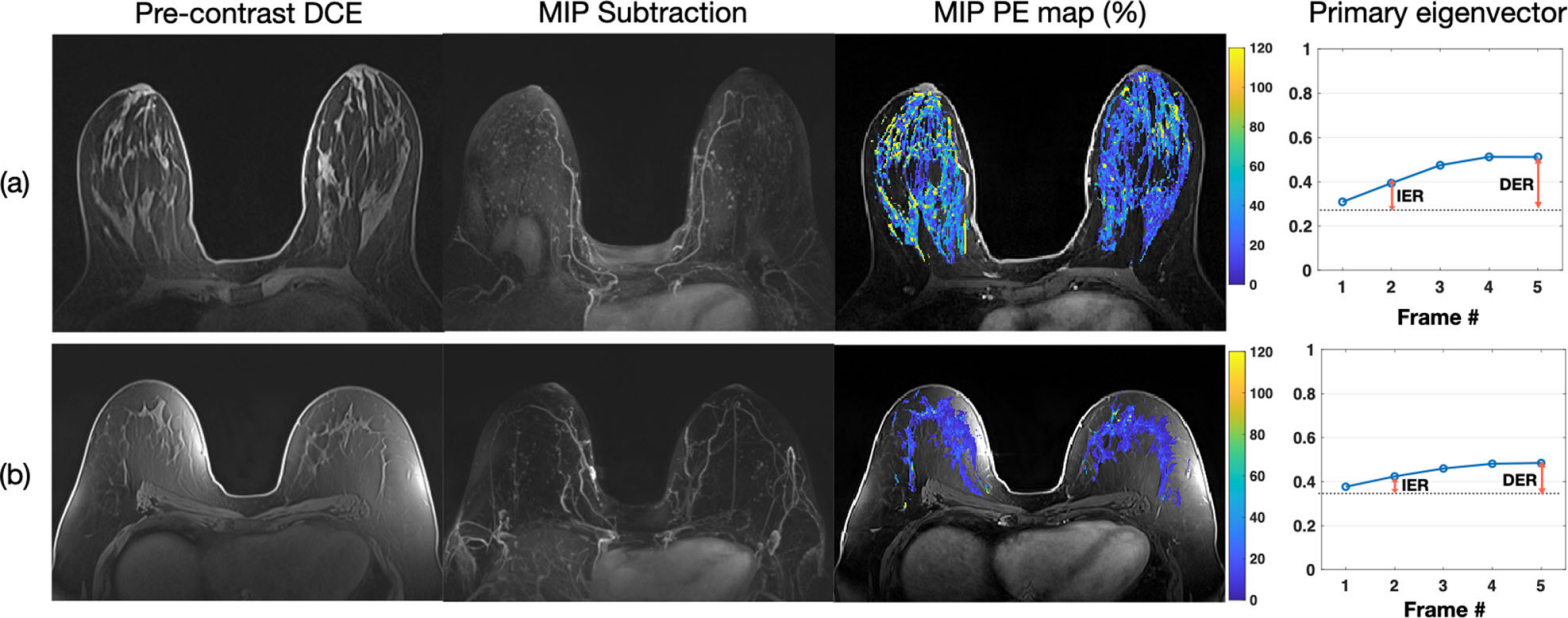
MRIs and BPE of two representative nondense-breast overweight/obesity postmenopausal women with (**a**) high-risk non-*BRCA* mutation and (**b**) *BRCA* mutation. MRI images include precontrast DCE-MRI, MIP of first postcontrast subtraction DCE-MRI, MIP of ten slices of PE map, and the primary eigenvector used to measure IER and DER. **a** A 53-year-old woman with a lifetime risk of 22.2% and a BMI of 25.12. FGT level is scattered fibroglandular tissue, and BPE level is marked. **b** A 48-year-old *BRCA1*-positive woman with a BMI of 31.46. FGT level is scattered fibroglandular tissue, and BPE level is mild

The comparison of women stratified by breast density is shown in Table 3. In dense-breast women, significant differences in PE_FGT_, PE_Breast_, IER, and DER exist between the high-risk non-*BRCA* and nonhigh-risk groups. Figure 4 displays example images of two dense-breast women. The high-risk non-*BRCA* woman (Fig. 4a) has higher PE_FGT_, PE_breast_, IER, and DER than the nonhigh-risk woman (Fig. 4b). In nondense-breast women, the difference in PE_Breast_ between high-risk non-*BRCA* and *BRCA* groups is significant (0.6 vs. 0.4%, adjusted *p* = 0.096). Figure 5 presents example images of two nondense-breast women. The high-risk non-*BRCA* woman (Fig. 5a) has higher PE_Breast_ than the *BRCA1-positive* woman (Fig. 5b).

**Table 3:** Quantitative BPE comparison in dense-breast and nondense-breast women using Mann–Whitney U-test

	High-risk non-*BRCA*	Nonhigh-risk	Adjusted *p*	High-risk non-*BRCA*	*BRCA*	Adjusted *p*
**Dense-breast**	*N* = 212	*N* = 40		*N* = 212	*N* = 100	
PE_FGT_ (%) (Median [IQR])	10.4 (18.5)	6.3 (8.1)	0.012*	10.4 (18.5)	8.4 (11.7)	0.288
PE_breast_ (%) (Median [IQR])	2.5 (4.7)	1.0 (1.2)	< 0.001*	2.5 (4.7)	1.9 (3.3)	0.582
SER_FGT_ (%) (Median [IQR])	7.6 (6.9)	9.9 (6.9)	0.108	7.6 (6.9)	9.5 (7.2)	0.108
SER_breast_ (%) (Median [IQR])	1.8 (2.1)	1.5 (2.1)	1.0	1.8 (2.1)	2.0 (2.9)	0.714
IER (%) (Median [IQR])	35.2 (59.8)	24.1 (29.3)	0.024*	35.2 (59.8)	28.3 (37.5)	0.276
DER (%) (Median [IQR])	97.0 (114.3)	62.5 (52.1)	0.006*	97.0 (114.3)	74.5 (98.5)	0.288
**Nondense-breast**	*N* = 87	*N* = 31		*N* = 87	*N* = 65	
PE_FGT_ (%) (Median [IQR])	9.2 (10.5)	11.2 (8.1)	1.0	9.2 (10.5)	7.5 (8.8)	0.624
PE_breast_ (%) (Median [IQR])	0.6 (0.9)	0.6 (0.7)	1.0	0.6 (0.9)	0.4 (0.7)	0.096*
SER_FGT_ (%) (Median [IQR])	6.9 (5.3)	11.0 (7.7)	0.132	6.9 (5.3)	8.4 (7.5)	0.804
SER_breast_ (%) (Median [IQR])	0.5 (0.5)	0.4 (0.9)	1.0	0.5 (0.5)	0.4 (0.5)	1.0
IER (%) (Median [IQR])	29.6 (33.4)	28.8 (30.2)	1.0	29.6 (33.4)	27.8 (24.7)	0.882
DER (%) (Median [IQR])	77.1 (61.0)	72.3 (41.1)	1.0	77.1 (61.0)	73.8 (45.1)	0.804

*Significant difference was identified as adjusted *p* value < 0.10 (pre-specified threshold of FWER)

Note: number of hypothesis testing = 6, adjusted *p* value = unadjusted *p* value / 6

The comparison of women stratified by menopausal status is demonstrated in Supplementary Material S3. We found no significant differences in both the premenopausal subcohort and postmenopausal subcohort.

### Correlation analysis of quantitative BPE

In the univariate analysis, age and menopausal status significantly correlate with all BPE measurements (Supplementary Material S4). However, only menopausal status significantly correlates with all BPE measurements in the multivariable analysis (Supplementary Material S5). With the identified difference for PE_breast_ comparison based on the smallest *p* value among the comparison for six measures in Table 2, linear regression results of PE_breast_ was further assessed in Table 4. Both univariate and multivariable analyses of PE_breast_ found significant correlations with age, BMI, menopausal status, and FGT level. Specifically, higher PE_breast_ was correlated with younger age, lower BMI, premenopausal status, and higher FGT levels.

**Table 4:** Univariate and multivariable linear regression analysis for PE_breast_ increase in all women

	Univariate linear regression analysis		Multivariable linear regression analysis	
	Coefficient (CI)	*p* value	Coefficient (CI)	*p* value
Age	−0.222 (−0.275, −0.169)	< 0.001*	−0.101 (−0.181, −0.021)	0.014*
BMI	−0.232 (−0.296, −0.169)	< 0.001*	−0.111 (−0.181, −0.041)	0.002*
Menopausal status	−0.084 (−0.104, −0.064)	< 0.001*	−0.033 (−0.061, −0.005)	0.022*
Hormonal treatment	0.023 (−0.0, 0.046)	0.052	−0.002 (−0.023, 0.019)	0.860
FGT level	0.172 (0.139, 0.206)	< 0.001*	0.095 (0.054, 0.136)	< 0.001*
BRCA gene mutation	−0.016 (−0.038, 0.007)	0.167	−0.012 (−0.033, 0.009)	0.255

*Significant difference with unadjusted *p* value < 0.05

### Comparisons of quantitative BPE after PSM

The clinical and radiographic characteristics of the patient cohort after PSM are summarized in Supplementary Material S6. There were no significant differences in age, BMI, menopause status, hormone treatment history, and FGT level among matched groups. Table 5 shows a quantitative BPE comparison after PSM. We found no significant differences between the high-risk non-*BRCA* and nonhigh-risk groups in all BPE measurements. However, significant differences were observed between high-risk non-*BRCA* and *BRCA* groups in PE_FGT_ (11.5 vs. 8.0%, adjusted *p* = 0.018) and SER_FGT_ (7.2% vs. 9.3%, adjusted *p* = 0.066).

**Table 5:** Quantitative BPE comparison in all women using Mann–Whitney U−test after propensity score matching using age, BMI, menopausal status, hormonal treatment history within six months before MRI, and FGT level

	High-risk non-*BRCA*	Nonhigh-risk	Adjusted *p*	High-risk non-*BRCA*	*BRCA*	Adjusted *p*
PE_FGT_ (%) (Median [IQR])	8.3 (13.6)	8.2 (8.0)	1.0	11.5 (17.0)	8.0 (9.8)	0.018*
PE_Breast_ (%) (Median [IQR])	0.7 (1.8)	0.8 (0.9)	1.0	1.4 (3.7)	1.0 (2.2)	0.192
SER_FGT_ (%) (Median [IQR])	8.3 (7.2)	10.2 (7.2)	0.828	7.2 (6.5)	9.3 (7.6)	0.066*
SER_Breast_ (%) (Median [IQR])	0.8 (1.4)	1.1 (1.7)	0.636	1.0 (1.7)	1.1 (2.2)	1.0
IER (%) (Median [IQR])	31.1 (38.3)	26.4 (30.2)	1.0	34.2 (46.8)	28.2 (31.2)	0.174
DER (%) (Median [IQR])	76.1 (69.6)	68.4 (48.2)	1.0	84.6 (85.0)	73.9 (71.1)	0.156

*Significant difference was identified as adjusted *p* value < 0.10 (pre-specified threshold of FWER)

Note: number of hypothesis testing = 6, adjusted *p* value = unadjusted *p* value / 6

### Analyses of enhancement thresholds and phase selection for BPE quantification

**Fig. 6:**
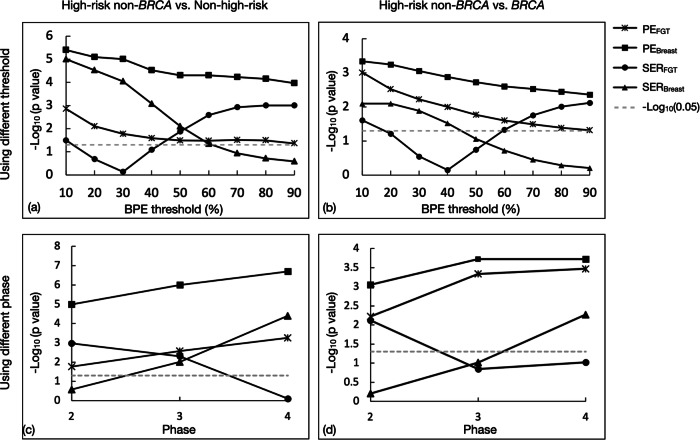
Manhattan plot of the unadjusted value profiles of Mann–Whitney U-test comparing four quantitative BPE measurements, PE_FGT_, PE_Breast_, SER_FGT_, and SER_Breast_, computed by using (**a**, **b**) a wide range of BPE enhancement threshold and (**c**, **d**) different postcontrast phases as the early phase. A reference line with a *p* of 0.05 is shown as the gray dashed line. The data point above the reference line indicated a significant difference

The Manhattan plots in Fig. 6 present the unadjusted *p* values for quantitative BPE comparisons using varying PE, SER enhancement thresholds, and postcontrast phases. We observed significant differences in both comparisons for PE_FGT_ and PE_breast_ across all thresholds (10–90%) and all three postcontrast phases. SER_FGT_ was significantly different using thresholds between 60 and 90% and using the second phase, while SER_breast_ was significantly different using thresholds between 10 and 40% and using the fourth phase in both comparisons.

## Discussion

Our study demonstrated a difference in quantitative BPE among different groups stratified by lifetime breast cancer risk and *BRCA* germline mutation status. Specifically, BPE is higher for the high-risk non-*BRCA* group than for the nonhigh-risk group, especially in the dense-breast subcohort. More importantly, BPE is higher for the high-risk non-*BRCA* group than for the *BRCA* group, especially in the nondense-breast subcohort. Linear regression analysis shows that factors significantly affecting BPE include age, BMI, menopausal status, and FGT level. After adjusting for these confounding factors, the difference in BPE between *BRCA* carriers and noncarriers can still be observed.

There is ongoing controversy regarding the relationship between BPE and various lifetime risk factors. In our study, PE_breast_ is significantly associated with age, BMI, menopausal status, and FGT level. BPE is known to be hormone-sensitive, which could explain the potential reason for the impact of age and menopausal status on BPE [23, 24, 31]. Previous studies reported an association between higher BMI and higher qualitative BPE [31, 32], while the underlying mechanism is not fully understood. A possible explanation is that adipose tissue can serve as a significant source of estrogen [33]. In our analysis, however, BMI is inversely correlated with quantitative BPE, possibly due to differences in patient selection and BPE quantification methods. Besides, the positive correlation between FGT and BPE in our study is consistent with previous literature [34, 35].

Prior studies of qualitative and quantitative BPE have shown that high-risk women with higher BPE had a higher chance of breast cancer [15, 36]. Compared with previous studies that correlated BPE with cancer development and mainly focused on high-risk patients, our study included women without a high lifetime breast cancer risk. According to our findings, individuals with high lifetime risk at baseline tended to have higher BPE than those without before adjusting for confounding factors. This finding may be attributed to the difference in known factors, like age, menopausal status, and FGT levels, since the differences in BPE disappeared after adjusting for these confounding factors.

The results of our study in the BPE comparison of *BRCA1/2* mutation carriers and noncarriers in high-risk patients are consistent with previous studies [18, 21], which found that *BRCA* mutation carriers had lower BPE than noncarriers. *BRCA* patients (assumed to be at the highest risk for developing breast cancer compared to everyone else) do not necessarily have the highest levels of BPE. After accounting for other potential influencing variables using PSM, these two groups have residual BPE differences. These findings may suggest that the *BRCA* germline mutation may affect quantitative BPE. Further investigation into the biological underpinnings of these effects is essential for leveraging quantitative BPE in breast cancer risk stratification.

In our study, we found that PE_FGT_ and PE_breast_ showed consistent differences between different groups across a wide range of intensity enhancement ratio thresholds and postcontrast phases. Currently, there is no standardized approach for determining this threshold value and selecting this postcontrast phase in the BPE quantification process. The consistent results that we observed suggest the potential robustness of BPE measurement as a biomarker correlated with breast cancer lifetime risk.

Our study has some limitations. One limitation is that vessels are challenging to visualize in dense breasts with MRI. Some vessels were likely counted as FGT during the segmentation procedure, resulting in a little overestimation of FGT and BPE. Additionally, due to the limited number of patients, the inclusion of patients with germline mutations other than *BRCA1/2*, such as *TP53*, *STK11*, and *ATM*, as a separate group was not feasible. However, these gene mutations are less common, and there is still much to learn about them. The absence of short-term MRI follow-up might be a limitation in confirming the complete absence of breast cancer due to the retrospective nature of the study. However, our stringent screening process reduced the likelihood of including patients with breast cancer. Finally, to assess if this study may have wide clinical use, it is necessary to undertake more research in a prospective environment for extended validation.

In conclusion, our study reveals that quantitative BPE measures are associated with lifetime breast cancer risk in non-*BRCA* mutation carriers and *BRCA* germline mutation status in high-risk women. These associations have been attributed to the presence of several lifetime risk factors. Differences in quantitative BPE between *BRCA* mutation carriers and high-risk noncarriers persisted after adjusting for known factors. Our work provides a potential explanation for the cancer pathophysiological mechanisms underlying the lifetime risk model from the perspective of BPE. In the future, additional research is necessary to determine if quantitative BPE can function as an independent risk factor enhancing breast cancer risk stratification.

## Supplementary Information

Supplementary Material

## Abbreviations

BPE: Background parenchymal enhancement
DCE: Dynamic contrast-enhanced
DER: Delayed enhancement ratio
FGT: Fibroglandular tissue
FWER: Family-wise error rate
IER: Initial enhancement ratio
IQR: Interquartile range
MIP: Maximum-intensity projection
PE: Percent enhancement
PSM: Propensity score matching
SER: Signal enhancement ratio
T1-NFS: T1-weighted non-fat-suppressed
