# Supplementary material for: Quantitative assessment of background parenchymal enhancement is associated with lifetime breast cancer risk in screening MRI
Source: Eur Radiol. 2024 Apr 29;34(10):6358–68. doi: 10.1007/s00330-024-10758-9 (PMC11399191; doi:10.1007/s00330-024-10758-9)
Supplement: Supplementary file 1 — Supplementary Material [file 330_2024_10758_MOESM1_ESM.pdf]

Quantitative assessment of background parenchymal enhancement is associated with lifetime breast cancer risk in screening MRI

Electronic Supplementary Material (ESM)

Eur Radiol (2024) Yan R, Murakami W, Mortazavi S et al.

Supplementary Material S1. MRI Imaging Parameters

	T1-NFS	DCE-MRI
matrix	448 × 448 x 176	448 × 448 x 176
field of view (FOV)	380 mm x 380 mm	380 mm x 380 mm
slice thickness	1.1 mm	1.1 mm
flip angle	20°	10°
repetition time (TR)	6.62 msec	4.14 msec
echo time (TE)	2.91 msec	1.54 msec

Supplementary Material S2. Four Quantitative BPE Calculations

The percent enhancement (PE) at each voxel, $\vec{r}$, representing wash-in enhancement characteristics was measured as follows:

$$\mathrm{PE}(\vec{\mathrm{r}})(\%) = \left(\frac{\mathrm{I_{early}}(\vec{\mathrm{r}}) - \mathrm{I_{pre}}(\vec{\mathrm{r}})}{\mathrm{I_{pre}}(\vec{\mathrm{r}})}\right) \times 100 \tag{1}$$

where I_{pre} is pre-contrast image intensity, and I_{early} is 120-second early-phase post-contrast image intensity.

The signal enhancement ratio (SER) at each voxel representing delayed enhancement characteristics was measured as follows:

$$\mathrm{SER}(\vec{\mathrm{r}})(\%) = \left(\frac{\mathrm{I_{early}}(\vec{\mathrm{r}}) - \mathrm{I_{pre}}(\vec{\mathrm{r}})}{\mathrm{I_{delayed}}(\vec{\mathrm{r}}) - \mathrm{I_{pre}}(\vec{\mathrm{r}})}\right) \times 100 \tag{2}$$

where I_{pre} is pre-contrast image intensity, and I_{early} is 120-second early-phase post-contrast image intensity, and $I_{delayed}$ is 390-second delayed-phase post-contrast image intensity.

The segmented masks of the whole breast and FGT are denoted as M_{Breast} and M_{FGT}. We identified voxels within the FGT region with enhancement ratios at or above a predetermined enhancement ratio threshold. The total volume (in cm^3) of these voxels, denoted as V_{PE} in the PE map and V_{SER} in the SER map, was computed as follows:

$$\mathrm{V_{PE}} = \sum_{\vec{\mathrm{r}} \in \mathrm{M_{FGT}}} (\mathrm{PE}(\vec{\mathrm{r}}) \geq \mathrm{PE_T}) \times \mathrm{vol} \tag{3}$$

$$\mathrm{V_{SER}} = \sum_{\vec{\mathrm{r}} \in \mathrm{M_{FGT}}} (\mathrm{SER}(\vec{\mathrm{r}}) \geq \mathrm{SER_T}) \times \mathrm{vol} \tag{4}$$

where PE_T is the enhancement ratio threshold for PE at each voxel, SER_T is the enhancement ratio threshold for the SER at each voxel, and vol is voxel volume in cm^3. PE_T and SER_T were set as 30% and 90%, respectively.

Based on the segmentation masks M_{Breast} and M_{FGT}, the total volumes (cm^3) of FGT and the whole breast are computed as follows:

$$\mathrm{V_{FGT}} = \sum_{\vec{\mathrm{r}} \in \mathrm{M_{FGT}}} (\vec{\mathrm{r}}) \times \mathrm{vol} \tag{5}$$

$$\mathrm{V_{Breast}} = \sum_{\vec{\mathrm{r}} \in \mathrm{M_{Breast}}} (\vec{\mathrm{r}}) \times \mathrm{vol} \tag{6}$$

Finally, we estimated BPE over the FGT volume (FGT-wise BPE) and the whole breast volume (breast-wise BPE) to generate four percentage-based BPE measures as follows:

$$\mathrm{PE_{FGT}}(\%) = \frac{\mathrm{V_{PE}}}{\mathrm{V_{FGT}}} \times 100 \tag{7}$$

$$\mathrm{PE_{Breast}}(\%) = \frac{\mathrm{V_{PE}}}{\mathrm{V_{Breast}}} \times 100 \tag{8}$$

$$\mathrm{SER_{FGT}}(\%) = \frac{\mathrm{V_{SER}}}{\mathrm{V_{FGT}}} \times 100 \tag{9}$$

$$\mathrm{SER_{Breast}}(\%) = \frac{\mathrm{V_{SER}}}{\mathrm{V_{Breast}}} \times 100 \tag{10}$$

Supplementary Material S3. Quantitative BPE comparison in premenopausal and postmenopausal women using Mann–Whitney U-test

	High-risk non-*BRCA*	Non-high-risk	Adjusted *p*	High-risk non-*BRCA*	*BRCA*	Adjusted *p*
**Premenopausal women**	N = 198	N =19		N = 198	N = 91	
PE_{FGT} (%) (Median (IQR))	12.5 (17.7)	10.4 (9.9)	1.0	12.5 (17.7)	9.5 (12.5)	.298
PE_{Breast} (%) (Median (IQR))	2.6 (5.0)	1.0 (2.0)	.192	2.6 (5.0)	2.1 (3.2)	.838
SER_{FGT} (%) (Median (IQR))	6.7 (5.8)	5.5 (5.2)	1.0	6.7 (5.8)	8.1 (8.1)	.426
SER_{Breast} (%) (Median (IQR))	1.4 (2.2)	1.0 (1.3)	.678	1.4 (2.2)	1.7 (2.7)	1.0
IER (%) (Median (IQR))	43.8 (58.4)	31.7 (38.7)	1.0	43.8 (58.4)	35.1 (33.8)	.294
DER (%) (Median (IQR))	111.6 (111.9)	86.4 (52.0)	1.0	111.6 (111.9)	90.0 (71.5)	.271
**Postmenopausal women**	N = 101	N = 52		N = 101	N = 74	
PE_{FGT} (%) (Median (IQR))	7.1 (11.0)	7.3 (8.0)	1.0	7.1 (11.0)	5.9 (7.9)	1.0
PE_{Breast} (%) (Median (IQR))	0.7 (1.4)	0.6 (0.8)	1.0	0.7 (1.4)	0.5 (0.7)	.156
SER_{FGT} (%) (Median (IQR))	9.2 (6.9)	11.1 (6.1)	.114	9.2 (6.9)	10.1 (6.4)	1.0
SER_{Breast} (%) (Median (IQR))	0.9 (1.6)	1.1 (1.6)	1.0	0.9 (1.6)	0.7 (0.9)	1.0
IER (%) (Median (IQR))	26.8 (28.5)	25.4 (23.0)	1.0	26.8 (28.5)	22.6 (21.4)	1.0
DER (%) (Median (IQR))	61.7 (57.4)	58.1 (41.6)	1.0	61.7 (57.4)	54.3 (45.7)	1.0

BPE, background parenchymal enhancement; PE, percent enhancement; SER, signal enhancement ratio; IER, initial enhancement ratio; DER, delayed enhancement ratio; FGT, fibroglandular tissue; IQR, interquartile range.
* Significant different with adjusted *p* value < .10

Supplementary Material S4. Univariate linear regression analysis for Quantitative BPE increases.

	PE_{FGT}		PE_{Breast}		SER_{FGT}	
	Coefficient (CI)	*p* value	Coefficient (CI)	*p* value	Coefficient (CI)	*p* value
Age	-0.173 [-0.237, -0.108]	<0.001*	-0.222 [-0.275, -0.169]	<0.001*	0.089 [0.03, 0.147]	0.003*
BMI	-0.027 [-0.105, 0.051]	0.495	-0.232 [-0.296, -0.169]	<0.001*	-0.118 [-0.187, -0.049]	<0.001*
Menopausal status	-0.07 [-0.094, -0.046]	<0.001*	-0.084 [-0.104, -0.064]	<0.001*	0.055 [0.034, 0.077]	<0.001*
Hormonal treatment	0.034 [0.007, 0.061]	0.013*	0.023 [-0.0, 0.046]	0.052	0.001 [-0.023, 0.025]	0.948
FGT level	0.042 [-0.0, 0.085]	0.051	0.172 [0.139, 0.206]	<0.001*	0.027 [-0.011, 0.065]	0.168
BRCA gene mutation	-0.029 [-0.055, -0.003]	0.029*	-0.016 [-0.038, 0.007]	0.167	0.026 [0.002, 0.049]	0.030*

	SER_{Breast}		IER		DER	
	Coefficient (CI)	*p* value	Coefficient (CI)	*p* value	Coefficient (CI)	*p* value
Age	-0.215 [-0.279, -0.151]	<0.001*	-0.078 [-0.117, -0.039]	<0.001*	-0.159 [-0.212, -0.106]	<0.001*
BMI	-0.393 [-0.464, -0.322]	<0.001*	-0.044 [-0.09, 0.003]	0.067	-0.047 [-0.112, 0.017]	0.148
Menopausal status	-0.052 [-0.076, -0.028]	<0.001*	-0.038 [-0.052, -0.023]	<0.001*	-0.073 [-0.092, -0.053]	<0.001*
Hormonal treatment	0.031 [0.004, 0.058]	0.025*	0.013 [-0.004, 0.029]	0.126	0.025 [0.003, 0.047]	0.028*
FGT level	0.277 [0.241, 0.313]	<0.001*	0.047 [0.021, 0.072]	<0.001*	0.079 [0.044, 0.114]	<0.001*
BRCA gene mutation	0.011 [-0.015, 0.038]	0.405	-0.01 [-0.026, 0.006]	0.223	-0.016 [-0.038, 0.005]	0.139

Supplementary Material S5. Multivariate linear regression analysis for Quantitative BPE increases.

	PE_{FGT}		PE_{Breast}		SER_{FGT}	
	Coefficient (CI)	*p* value	Coefficient (CI)	*p* value	Coefficient (CI)	*p* value
Age	-0.094 [-0.196, 0.008]	0.071	-0.101 [-0.181, -0.021]	0.014*	-0.004 [-0.095, 0.087]	0.924
BMI	0.009 [-0.081, 0.099]	0.842	-0.111 [-0.181, -0.041]	0.002*	-0.124 [-0.204, -0.044]	0.002*
Menopausal status	-0.044 [-0.08, -0.008]	0.018*	-0.033 [-0.061, -0.005]	0.022*	0.067 [0.035, 0.099]	<0.001*
Hormonal treatment	0.023 [-0.005, 0.05]	0.102	-0.002 [-0.023, 0.019]	0.860	0.0 [-0.024, 0.024]	0.995
FGT level	-0.012 [-0.065, 0.041]	0.654	0.095 [0.054, 0.136]	<0.001*	0.033 [-0.014, 0.08]	0.165
BRCA gene mutation	-0.036 [-0.062, -0.009]	0.008*	-0.012 [-0.033, 0.009]	0.255	0.029 [0.006, 0.053]	0.015*

	SER_{Breast}		IER		DER	
	Coefficient (CI)	*p* value	Coefficient (CI)	*p* value	Coefficient (CI)	*p* value
Age	-0.157 [-0.243, -0.07]	<0.001*	-0.001 [-0.063, 0.061]	0.979	-0.019 [-0.103, 0.065]	0.656
BMI	-0.197 [-0.273, -0.121]	<0.001*	-0.0 [-0.055, 0.054]	0.990	0.033 [-0.041, 0.106]	0.380
Menopausal status	0.039 [0.009, 0.07]	0.012*	-0.032 [-0.054, -0.01]	0.005*	-0.058 [-0.088, -0.029]	<0.001*
Hormonal treatment	-0.004 [-0.027, 0.019]	0.752	0.007 [-0.009, 0.024]	0.398	0.015 [-0.008, 0.037]	0.200
FGT level	0.209 [0.164, 0.254]	<0.001*	0.025 [-0.007, 0.057]	0.125	0.044 [0.001, 0.088]	0.044*
BRCA gene mutation	0.018 [-0.005, 0.04]	0.118	-0.008 [-0.024, 0.008]	0.339	-0.015 [-0.037, 0.006]	0.166

BMI, body mass index; BPE, background parenchymal enhancement; PE, percent enhancement; SER, signal enhancement ratio; IER, initial enhancement ratio; DER, delayed enhancement ratio; FGT, fibroglandular tissue; CI, confidence interval.
* Significant different with adjusted p value < .05

Supplementary Material S6. Patient characteristics after propensity score matching.

	High-risk non-*BRCA*	Non-high-risk	Unadjusted *p*	High-risk non-*BRCA*	*BRCA*	Unadjusted *p*
**No. of patients**	71	71		165	165	
**Age (median (range))**	55 (31-76)	56 (30-86)	.455	44 (23-70)	40 (21-83)	.189
**BMI (median (range))**	25.42 (17.39-39.62)	24.1 (18.7-40.7)	.143	24.75 (17.26-48.71)	25.1 (17.4-44.4)	.389
**Menopausal status**			1.0			.912
Premenopausal	18 (25.4%)	19 (26.8%)		89 (53.9%)	91 (55.2%)	
Postmenopausal	53 (74.6%)	52 (73.2%)		76 (46.1%)	74 (44.8%)	
**Hormone treatment**			1.0			.906
Yes	24 (33.8%)	23 (32.4%)		51 (30.9%)	53 (32.1%)	
No	47 (66.2%)	48 (67.6%)		114 (69.1%)	112 (67.9%)	
**FGT**			.69			.804
Almost entirely fat	7 (9.9%)	4 (5.6%)		7 (4.2%)	10 (6.1%)	
Scattered	30 (42.3%)	27 (38.0%)		61 (37.0%)	55 (33.3%)	
Heterogeneous	27 (38.0%)	32 (45.1%)		57 (34.5%)	61 (37.0%)	
Extreme	7 (9.9%)	8 (11.3%)		40 (24.2%)	39 (23.6%)	
**BPE**			.006*			< .001*
Minimal	22 (31.0%)	34 (47.9%)		38 (23.0%)	72 (43.6%)	
Mild	25 (35.2%)	17 (23.9%)		52 (31.5%)	55 (33.3%)	
Moderate	16 (22.5%)	20 (28.2%)		53 (32.1%)	33 (20.0%)	
Marked	8 (11.3%)	0 (0.0%)		22 (13.3%)	5 (3.0%)	

BMI, body mass index; BPE, background parenchymal enhancement; FGT, fibroglandular tissue.
The *p* values are from Mann-Whitney U-test for age and BMI, and chi-squared test for menopausal status, hormone treatment history, FGT and BPE.
* Significant different with unadjusted *p* value < .05
